# Effect of Attitudinal, Situational and Demographic Factors on Annoyance Due to Environmental Vibration and Noise from Construction of a Light Rapid Transit System

**DOI:** 10.3390/ijerph13121237

**Published:** 2016-12-14

**Authors:** Daniel Wong-McSweeney, James Woodcock, David Waddington, Eulalia Peris, Zbigniew Koziel, Andy Moorhouse, María Dolores Redel-Macías

**Affiliations:** 1 Acoustics Research Centre, University of Salford, Salford M5 4WT, UK.; J.S.Woodcock@salford.ac.uk (J.W.); d.c.waddington@salford.ac.uk (D.W.); e.peris@salford.ac.uk (E.P.); zbigniew.koziel@manchester.ac.uk (Z.K.); a.t.moorhouse@salford.ac.uk (A.M.); 2 Department of Rural Engineering, University of Córdoba, 14071 Córdoba, Spain; ig1remam@uco.es

**Keywords:** construction, vibration, LRT, annoyance, attitudinal, situational, demographic

## Abstract

The aim of this paper is to determine what non-exposure factors influence the relationship between vibration and noise exposure from the construction of a Light Rapid Transit (LRT) system and the annoyance of nearby residents. Noise and vibration from construction sites are known to annoy residents, with annoyance increasing as a function of the magnitude of the vibration and noise. There is not a strong correlation between exposure and levels of annoyance suggesting that factors not directly related to the exposure may have an influence. A range of attitudinal, situational and demographic factors are investigated with the aim of understanding the wide variation in annoyance for a given vibration exposure. A face-to-face survey of residents (*n* = 350) near three sites of LRT construction was conducted, and responses were compared to semi-empirical estimates of the internal vibration within the buildings. It was found that annoyance responses due to vibration were strongly influenced by two attitudinal variables, concern about property damage and sensitivity to vibration. Age, ownership of the property and the visibility of the construction site were also important factors. Gender, time at home and expectation of future levels of vibration had much less influence. Due to the measurement methods used, it was not possible to separate out the effects of noise and vibration on annoyance; as such, this paper focusses on annoyance due to vibration exposure. This work concludes that for the most cost-effective reduction of the impact of construction vibration and noise on the annoyance felt by a community, policies should consider attitudinal factors.

## 1. Introduction

It has previously been reported that increases in noise and vibration exposure lead to increased levels of annoyance in residential environments [1,2,3,4,5]. The relationship between exposure and annoyance is, however, weak, suggesting that other factors need to be taken into account [6]. Baseline models of human responses to vibration have been established [7], and this paper is intended to extend these models by looking at the influence of mediating and moderating factors, herein referred to as non-exposure factors. While there are relatively few investigations into the factors influencing the annoyance due to vibration, there have been many for the annoyance of noise. For noise annoyance, exposure explains less than 20% of variation [8,9], the remainder being explained by other factors, which can be divided into attitudinal, situational and demographic factors. The first of the attitudinal factors that may increase noise annoyance is noise sensitivity [9,10,11,12]. A difference of 11 dB in noise exposure is found by Miedema and Vos [13] to produce equivalent annoyance depending on noise sensitivity. Between 10% and 26% of the variance in reported annoyance can be explained by noise sensitivity [14,15]. It is suggested by Heinonen [16] that sensitivity is both a psychological and somatic response and perhaps has a genetic component.

Along with sensitivity, another attitudinal factor affecting noise annoyance is a fear of the noise source [10]. Miedema and Vos [13] find that for equivalent annoyance, fear reduces the required railway noise level by up to 19 dB. A caveat is that few people experience fear due to railways. A further attitudinal factor is people’s expectation of future levels of noise, though reported results are inconclusive [17,18,19]. Finally, people’s perceptions of their neighbourhood are found to influence the annoyance of noise [20,21] with an 18-dB difference in noise level potentially producing equivalent annoyance [22].

Alongside attitudinal factors, there are situational factors. These can include the presence of other signs of the noise source, for example dirt, dust, smells or light, which can also lead to an increase in annoyance [23,24]. Another situational factor is the type of location, i.e., rural or town locations, though contrasting results are reported. Bradley and Jonah [25] report annoyance being greater in towns, while Lercher and Kofler [12] report greater annoyance rurally. The effect of background noise is similarly reported with contrary results. Fields [26] finds it has no impact, while Klaeboe et al. [27,28] find that it does. The visibility of the noise source is also found to increase the annoyance it produces [29], and similarly, Pedersen and Larsman [30] find that being able to see wind turbines is likely to increase the annoyance of the noise they produce. Increasing the length of time spent at home, and therefore, the level of exposure, is found to increase the annoyance of a noise source [11,31]. Generally, socio-demographic factors are found to have little effect on noise annoyance [10,13]. Age is, however, found to affect noise annoyance with those of middle age found to be most highly annoyed [32] compared to younger and older people. Finally, whether people own or rent the residence also had an effect on reported annoyance with owners being more annoyed [13].

As for noise, an increase in the magnitude of vibration leads to an increase in reported annoyance [3,4,5,33]. Standards in this area focus on objective vibration exposure and variables that affect it, such as magnitude, duration and proximity. Physical descriptors of the vibration, e.g., frequency and duration, as well as accompanying noise also correlate with annoyance from vibrations [34,35]. Waddington et al. [1] assess sixty different vibration descriptors and six frequency weightings for how well they predict annoyance. It is found that no one descriptor is particularly better, but that frequency weighting is important for increasing the correlation between exposure and response. Fields and Walker [22] describe that in the absence of vibration measurements, distance, and therefore magnitude, is related to the reports of vibration in people’s homes, but that other factors are important. The Spearman correlation coefficient between a range of exposure predictors and annoyance is found [1] to be low, suggesting that other factors are important. For annoyance from rail-related vibration, it is found that only a quarter of the variance in reported annoyance can be accounted for by the exposure-response relationship [4]. Schomer and Neathammer [36] find that rattling, as a result of ground-borne vibration, can bias annoyance by 12 dB. Woodroof and Griffin [37] conclude that rather than the magnitude of vibration from railways, it is the number of trains per 24-h period that correlates with annoyance. Similarly Gidlof et al. [38] find that annoyance from railway vibration is increased in the presence of railway noise and vice versa. Gidlof et al. [38] also look at train-related factors affecting annoyance, specifically the number of trains and the presence of ground-borne vibration. It is found that there is an increase in annoyance for higher vibration velocities. There is a large interaction between vibration and noise in causing annoyance.

A large socio-vibration survey carried out in Norway [39] determined exposure-response relationships for road and rail sources. The exposure-response relationship, from questionnaires and semi-empirical internal vibration estimates (from within the buildings), was modelled using ordinal logit regression. This study did not specifically look at whether other factors (e.g., age, vibration duration or number of events) affects the relationship between vibration and annoyance. Fields [40] link an associated sense of danger from the vibration source with increased annoyance. Peris et al. [6] investigate the factors affecting the annoyance from railway vibration again separating the factors into three groups: attitudinal, situational and demographic factors. It is found that concern for property damage and an expectation of increased future vibration are two attitudinal factors that increase annoyance. The situational factors that increase the annoyance are the type of residential area, the length of time spent at home and the visibility of the railway. The only demographic factor that affects the annoyance is the age of the respondent with increased annoyance for middle-aged people, as is found for noise annoyance. With regard to annoyance from construction vibration, little research has been published. It is reported [1] that at a given vibration exposure, construction vibration is found to be more annoying than rail vibration.

This paper investigates non-exposure factors affecting the annoyance reported due to vibration from constructions sites and presents a further analysis of data collected in the study of Waddington et al. [1]. Following previous work [6] the factors investigated are divided into attitudinal, situational and demographic factors. In the following section, this paper will describe the collection of the exposure data and the response data. Then, in Section 3, the statistical analyses are outlined. In Section 4, the results are presented for the separate factors investigated. The results are then discussed in Section 5 before conclusions are drawn in Section 6.

## 2. Methodology

### 2.1. Study Design

The measurements were made in the northwest of the UK in 2010 during the construction of a Light Rapid Transit (LRT) system [1,41]. A light rapid transit system was chosen because construction follows a line along which a cycle of construction activities are repeated. This means that residents at one end of the line who would have experienced the full cycle of construction could be surveyed without introducing bias regarding the vibration survey. Meanwhile noise and vibration measurements of the full construction cycle could be made further down the line.

Sites were identified from online mapping services and then visited to determine if they were appropriate. Three sites were chosen that were at the correct stage of construction and were without sources of vibration other than the construction site. Surveys, consisting of face-to-face questionnaires with residents in their homes, were conducted in these locations, and there were a total of 350 responses.

### 2.2. Vibration Exposure

**Figure 1:**
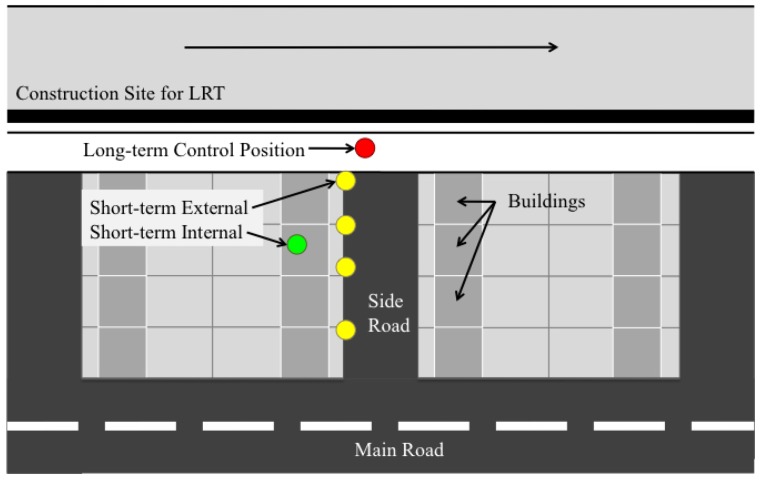
Diagram showing an overview of the construction vibration measurements setup.

A semi-empirical method was chosen to estimate the vibration exposure. Guralp CMD-5TD three axis force-accelerometers, time synchronised via GPS, were used to measure vibration at several locations, as shown in Figure 1. Estimation of the internal vibration was made using the Bornitz equation [42] based on both long-term (red dot) and short-term (yellow dots) measurements. The estimates were compared to an internal measurement (green dot) for validation. The difference in the measured and predicted values resulted in a relative error of 3.3 dB [1].

The locations characterised enabled the calculation of the 24-h internal vibration in 321 dwellings from the 350 in the sample. From the acceleration data, the Vibration Dose Value (VDV) was calculated. This metric was chosen because it is a vibration exposure descriptor outlined in the standard BS 5228.2:2009 “Code of practice for noise and vibration control on construction and open sites. Vibration”.

### 2.3. Noise Exposure

Although the focus of this work was exposure to environmental vibration, the evaluation of associated noise was performed to allow the consideration of combined effects from vibration and noise. Noise exposure from construction work at the most exposed external facade for each dwelling was calculated primarily from on-site measurements performed using the principles outlined in BS 5228-1:2009, which deals with stationary and mobile plants within closed areas or moving along a defined route. The factors considered are:

- sound power outputs of the processes and plant;
- periods of operation of the processes and plant;
- distance from sources and receivers;
- presence of shielding, screening and barriers;
- reflection of sound;
- soft ground attenuation;
- meteorological factors, if the distance between the source and receiver is greater than or equal to 50 m.

The standard also contains an extensive number of values for the sound power level of corresponding plants, which provides an opportunity to predict sound emission from machinery for which measurement results could not be obtained.

For the measurement of process noise source levels, a sound level meter was set up about 15 m away from sources or the route of a mobile source. The sound level meter was set at a distance of 1 m from the most exposed facade at a height above the ground of 1.5 m. The sound level meter maintained its position for all measurements over different days and was located in the vicinity of a long-term vibration monitor. The prediction of construction noise exposure was aided by the use of the long-term vibration measurements that were used to identify operations. All construction work took place between 07:00 and 19:00, and thus, $L_{day}$ is considered sufficient for expressing the noise exposure from construction sources [43].

### 2.4. Locations and Respondents

All three locations were in urban environments on the outskirts of a large city. At Locations A and C, the light-railway lines were being constructed in railway cuttings at the rear of properties, while at Location B, it was being constructed along a main road. Responses were collected from 350 residences across three locations, A (161 residences), B (124 residences) and C (65 residences), of which 133 respondents were male and 216 were female (1 not recorded). The range of ages covered by this sample was from 16–88, and Table 1 shows the distribution of respondents’ ages.

### 2.5. Questionnaire

The survey was carefully designed to separate out responses to vibration and to noise with different sections for each. It was pilot tested before being peer reviewed by five international experts in noise annoyance research [44]. The survey was conducted face-to-face to avoid any self-selection bias associated with postal questionnaires. Only one person per household was interviewed, specifically the person who answered the door unless it was a child under 16 years of age.

To avoid introducing bias about vibration, the questionnaire was introduced as a neighbourhood satisfaction survey. The first few sections were related to their satisfaction with both their neighbourhood and their home. With regard to annoyance, responses were characterised as the reported level of annoyance on a five-point semantic scale. Within the construction section, the activity was described as follows: “Construction activity, including demolition, piling, road works, drilling, surface activity such as bulldozers and loading trucks and any other construction activity”.

The noise annoyance question was laid out thusly: “Thinking about the time you have been living here, when indoors at home, how bothered, annoyed or disturbed have you been by hearing noise caused by [construction activity]? Would you say not at all (1), slightly (2), moderately (3), very (4) or extremely (5)?”.

Likewise the vibration annoyance question was laid out thusly: “Thinking about the time you have been living here, when indoors at home, how bothered, annoyed or disturbed have you been by feeling vibration or shaking or hearing or seeing things rattle, vibrate or shake caused by [construction activity]? Would you say not at all (1), slightly (2), moderately (3), very (4) or extremely (5)?”.

If a respondent reported not feeling vibration nor any effects of vibration (e.g., rattling), they were not then asked about where they felt it; what activities they were doing when there was vibration; nor how annoyed they were because of it. For these questions, they scored ‘0’, which was later recoded. An example of this was whether respondents were sensitive to vibration. If they had previously responded that they felt no vibration, they would not then be asked to rate their sensitivity to vibration (sensitivity), and a score of ‘0’ would be recorded. During the analysis of the data, those who had a score of ‘0’ for sensitivity were recoded to ‘1’ (not at all sensitive to vibration).

The questionnaire provided two routes into the vibration questions, either via noticing vibration or noticing rattling or shaking as a result of vibration. The questions that were skipped if neither vibration nor its effects were noticed were: sources of vibration; annoyance of vibration from different sources; expectation of future levels of vibration; concern for property damage; and sensitivity to vibration. Appendix A provides a summary of the recoded responses and what recoding was applied to the data after collection.

As described in Section 1, three categories of factors that potentially influenced people’s annoyance responses were used: attitudinal, situational and demographic. The specific factors are described here.

Sensitivity to vibration: A five-point semantic scale was used to measure the response from the question: “How sensitive would you say you are personally to vibration in general? Would you say you are not at all sensitive, slightly sensitive, moderately sensitive, very sensitive or extremely sensitive?” with the categories scoring 1–5, respectively.

Concern for property damage (concern): Again, a five-point semantic scale was used for this factor with the question: “We would like to know if you are concerned that the vibration may damage this home or your possessions inside it in any way. Are you, not at all concerned, slightly concerned, moderately concerned, very concerned or extremely concerned?”

Property ownership: A categorical scale was used for this factor with the question: “Looking at this list, which best describes your current situation with this home? Do you or your family: Own outright or with a mortgage, Part-rent and part-own with a mortgage, Rent from a private landlord/letting agency, Rent from a Housing Association or Council, Other.” Afterwards as part of the analysis these results were divided into those with some stake in the house (own or part-own) and those who did not (renting at all).

Visibility of the construction site: This was ascertained by asking “From any room in this home, can you see:” and then construction activity is one of the yes/no options.

Expectation of future levels of vibration: A three-point scale was used to measure this factor via responses from the question: “In the future, do you think the level of vibration you experience whilst indoors at home will get worse, get better or remain the same?”. This was dichotomised into A, worse, and B, same or better.

Time spent at home during construction site hours: The number of hours for which the respondent was home was determined by asking which hours they were at home with the full 24 h divided into 3-h blocks. The question used was: “During a typical weekday, that is, Monday to Friday, what times are you usually at home? Are you at home between:” with the options: 06:01 and 09:00, 09:01 and 12:00, 12:01 and 15:00, 15:01 and 18:00, 18:01 and 21:00, 21:01 and 00:00, 00:01 and 03:00, 03:01 and 06:00. During the analysis, the hours outside of those during which the construction site was active, specifically 18:01–06:00, were discarded.

Neighbourhood and home satisfaction: The question used for measuring neighbourhood and home satisfaction was “Looking at this card, overall, how satisfied or dissatisfied are you personally with living in this neighbourhood? Would you say that you are very satisfied, satisfied, neither satisfied nor dissatisfied, dissatisfied or very dissatisfied?”

Age and gender: Respondents were asked their age with the open question: “Do you mind me asking how old you are?” and their gender was recorded by the interviewer.

## 3. Statistical Analyses

The programme SPSS (SPSS Statistics v.20, IBM, Armonk, NY, USA) was used to archive and analyse the survey data. Following the work of Peris et al. [6] and Klaeboe et al. [39], an ordinal logistic regression was used to model the data and generate parameter estimates for each annoyance threshold (not at all, slightly annoyed, moderately, very and extremely annoyed). Putting these parameters into Equation (1) indicates the probability of having a response equal to or greater than *j*.

(1) $$\begin{array}{c} P\left(Y\geq j|X_{i}-x_{i}\right)=1-\left(\frac{e^{\tau_{j}-\beta x_{i}}}{1+e^{\tau_{j}-\beta x_{i}}}\right)\;\;\;\;\;\;\mathrm{where}\;j\in \left[1,...,J-1\right] \end{array}$$

where $\tau_{j}$ corresponds to the *j*-th estimated threshold and *β* is a vector of estimated parameters for both the exposure and the non-acoustic factors. *J* is the number of dependent variable categories and $X_{i}$ is a vector of independent variables containing firstly the exposure and then the modifying variable(s). For example, in applying this to the relationship between vibration exposure, concern for property damage and annoyance, $\tau_{j}$ are the annoyance thresholds; *β* would be a vector with the estimated coefficients relating the exposure to annoyance and the concern for property damage to annoyance. $x_{i}$ would be a vector of exposure values and modifying variables for an individual *i*. Two variables were included additively in each analysis, the vibration exposure and one of the non-acoustic factors.

The interactions between vibration exposure and each of non-acoustic factors were tested for moderation and mediation [45]. Moderation describes how the relationship between the descriptor and outcome variables can be amplified or nullified by a third variable. Mediation puts the third variable between the predictor and outcome variables such that it actually explains the relationship between them rather than merely increasing or decreasing it. For the mediation analysis, a comparison is made of the estimated coefficients with and without the extra factor being included [45], specifically the change of coefficients for the direct path without the extra factor (*c*) and the indirect path with the extra factor ($c^{\prime }$). The diagram in Figure 6, Section 4 shows an example of the comparison between the direct and indirect paths.

In using ordinal logistic regression, the assumption of proportional odds is implicitly made. The validity of this assumption was tested using the test of parallel lines, which compares the use of one set of coefficients over all thresholds to using different coefficients for each threshold. It was confirmed that indeed, the assumption of proportional odds was valid, as the chi-squared statistic for the general model (different thresholds) was rejected with significance greater than 0.1.

## 4. Results

### 4.1. Noise Exposure-Annoyance

**Figure 2:**
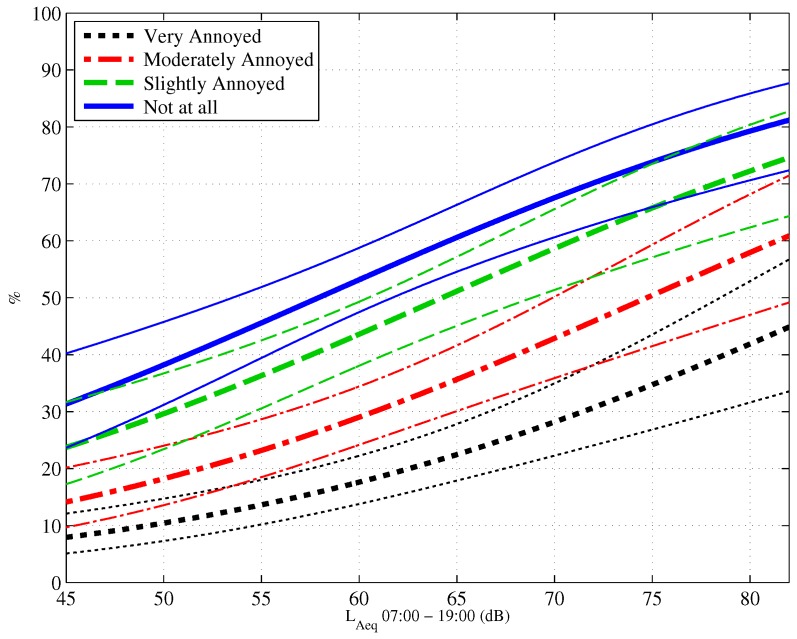
Exposure-response curves showing the proportion of people who are very annoyed, moderately annoyed, slightly annoyed and not at all annoyed by construction noise. The dashed lines represent 95% confidence intervals.

The exposure-response relationship showing the proportion of people reporting different degrees of annoyance due to noise for a given noise exposure from construction activities is shown in Figure 2. An ordinal logistic regression was used to derive the parameter estimates, and the dashed lines represent 95% confidence.

Because both noise and vibration exposure estimation procedures involve extrapolation from measurements at a reference point, there was a high degree of correlation between the estimates of noise and vibration exposure. This is evidenced by a Spearman’s correlation coefficient of 0.8 (p<0.01) between the two sets of exposures. This means that it is not possible to separate the annoyance due to construction noise from the annoyance due to construction vibration. Consequently, the following analyses examining the effect of non-exposure factors on annoyance are presented for construction vibration exposure, while the implications for future studies are discussed in Section 5.

### 4.2. Vibration Exposure-Annoyance

**Figure 3:**
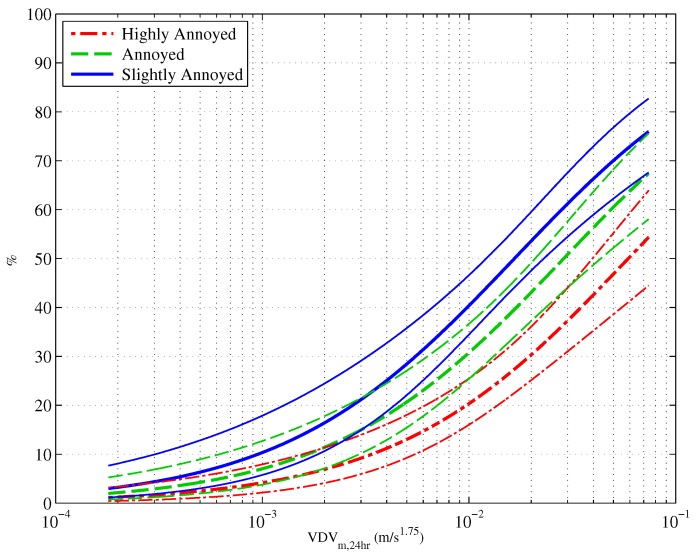
Exposure-response curves showing the proportion of people who are highly annoyed, annoyed and slightly annoyed by construction vibration. The dashed lines represent 95% confidence intervals. This figure is reproduced with permission from [7].

An ordinal logistic regression was used to model the relationship between construction vibration exposure, $\log_{10}$${\mathrm{VDV}}_{m,24h}$(${m/s}^{1.75}$), and construction vibration annoyance. This set a baseline against which the inclusion of other factors was compared. Figure 3 shows the proportion of people who reported being highly annoyed (HA), annoyed and slightly annoyed in response to construction vibration. Also shown on the graph are the 95% confidence intervals. Table 2 presents the parameter estimates for the exposure-response relationship between exposure and annoyance. The Cox and Snell $R_{\mathrm{pseudo}}^{2}$ value is approximately 0.17, against which later models are compared.

### 4.3. Self-Reported Sensitivity to Vibration

**Figure 4:**
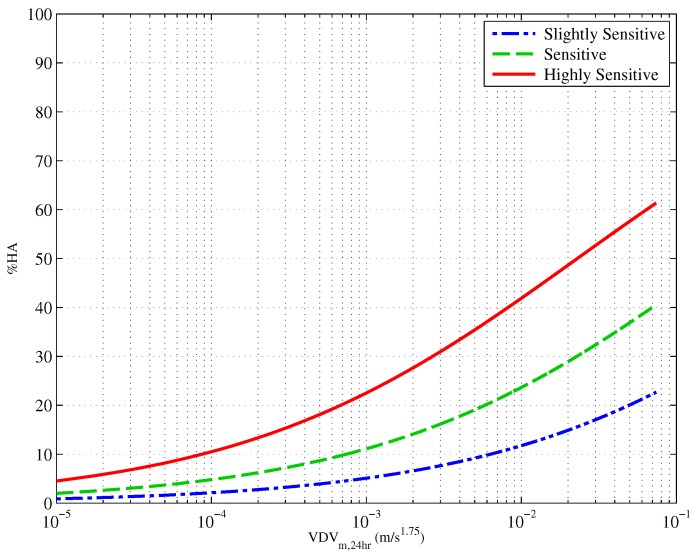
Exposure response relationship showing proportion of people reporting High Annoyance (%HA) at a given vibration exposure (VDV*_m, 24h_*), controlling for self-reported sensitivity to vibration. “Very sensitive” (blue) and “slightly sensitive” (red) to vibration. *n* = 250. Vibration data are “$W_{m}$” weighted.

An ordinal logistic regression was undertaken to predict annoyance using sensitivity and exposure as predictor variables, the results of which are presented in Table 3. Figure 4 presents the exposure-response curves from this analysis for those whose self-reported sensitivity to vibration was slightly sensitive, sensitive and highly sensitive. When sensitivity was included in the model, the Cox and Snell $R_{\mathrm{pseudo}}^{2}$ improved (0.316) compared to exposure alone (0.165). To determine whether there was an interaction of sensitivity with exposure, moderation was tested for the interaction term (exposure × sensitivity), and no significant interaction was found (p=0.501).

### 4.4. Concern for Property Damage

**Figure 5:**
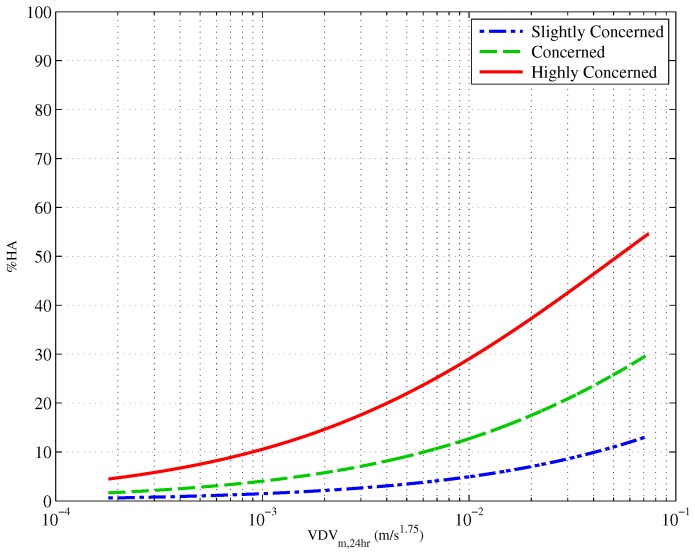
Exposure response for those highly annoyed by construction vibration controlling for levels property damage concern. *n* = 321. Vibration data are “$W_{m}$” weighted.

In the analysis using both exposure ($\log_{10}{\mathrm{VDV}}_{m,24h}$) and concern as predictors of annoyance, 29 cases were excluded for missing exposure data, and all of those respondents that previously reported feeling no vibration, and who therefore had a ‘0’ score for concern, were recoded as ‘1’, “not concerned”. The results of this regression are presented in Table 4. Figure 5 plots the exposure-response curves for annoyance from construction-vibration controlling for different levels of concern. In both the table and the graph, the values given are for the proportion of respondents reporting High Annoyance (%HA). Including concern as a predictor increased the Cox and Snell $R_{\mathrm{pseudo}}^{2}$ from 0.165 for exposure alone, to 0.471.

**Figure 6:**
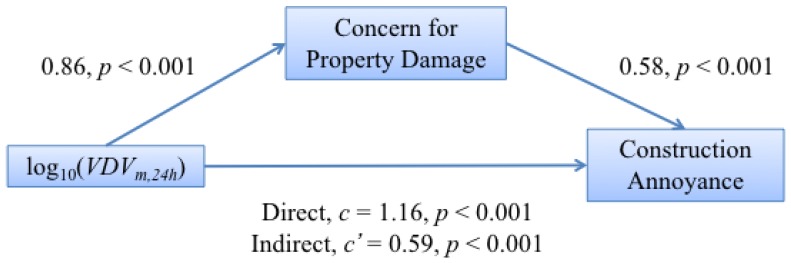
Schematic overview of the mediation model between vibration exposure, $\log_{10}{\mathrm{VDV}}_{m,24h}$, and annoyance via concern for property damage. The values given are the estimated coefficients linking the two variables and their *p*-value significance.

The interaction of vibration exposure and concern was tested for moderation using the interaction term exposure × concern. The result is a significant (p=0.068) moderating effect of increasing the annoyance when people were concerned. Mediation was tested, and this indicated that there was indeed an indirect effect of vibration on annoyance through concern for property damage. A schematic overview showing the model of mediation is presented in Figure 6 where it can be seen that the direct effect of exposure on annoyance has a coefficient of 1.16, which was reduced to 0.59 when concern was included. This suggests a partial mediation as the effect of exposure is not reduced to zero, and this in turn indicates that there are other mediating pathways linking exposure and vibration annoyance.

A Sobel test was carried out to determine whether the partial mediation is significant, and the *p*-value from this test was less than 0.001. The effect size $\kappa^{2}$, bounded by zero and one, was also calculated resulting in a value of $\kappa^{2}=0.211$ (CI (0.15 0.30)), indicating a large effect size [46].

### 4.5. Expectation

Table 5 presents the estimated parameters of the ordinal logit model for those highly annoyed (either “very” or “extremely”) and who expect the level of future vibrations to worsen. From this analysis, it is clear that there is a small increase in annoyance for those respondents who expect the future levels of vibration to worsen. As expectation was previously found to be an important factor for railways [47], further investigation was undertaken. Moderation was tested for the interaction exposure × expectation, but no significant (p=0.282) moderation was found.

### 4.6. Ownership

Table 6 presents the estimated parameters from the ordinal logit model for those reporting high annoyance from construction vibration controlling for ownership. Those who own or have a mortgage on their houses report being more annoyed by construction vibration than those who rent.

From an initial multivariate analysis, it was apparent that there was a potential connection between ownership and concern. An ordinal logistic regression was therefore used to find the relationship between ownership and concern, and Table 7 presents the resulting estimated parameters. Those who own or have mortgages on their houses are more concerned about property damage from vibration than those who rent. The Cox and Snell $R_{\mathrm{pseudo}}^{2}$ value increased from 0.112 for the exposure only model to 0.193 when ownership was included.

It has already been seen that to some extent, concern mediates the relationship between vibration and annoyance. Ownership was tested for moderation, and it was found that the interaction exposure × ownership had a significance of p=0.656, indicating that there is no significant moderation. From the test of mediation, it was found that while all coefficients were significant (p>0.1), the indirect path was smaller than the direct path ($c-c^{\prime }=0.56$), and the Sobel test had a *p*-value of p=0.066. This suggests that ownership also mediates the relationship between construction vibration and annoyance.

The mediation of ownership on the vibration exposure-concern relationship was also tested. All coefficients were found to be significant to better than p=0.1; the difference between the direct and indirect paths ($c-c^{\prime }$) was 0.11, and the Sobel test *p*-value was 0.042 for the mediation. This implies that ownership does indeed mediate the relationship between vibration exposure and concern. Therefore, ownership was found to influence the annoyance response, but that could be in part because ownership influences property damage concern, which in turn mediates the relationship between exposure and annoyance.

### 4.7. Visibility of the Construction Site

An ordinal regression was carried out with annoyance being predicted using both vibration exposure and visibility as predictor variables. Table 8 presents the estimated parameters from this analysis. The coefficients from this analysis were all significant (p<0.001), and inclusion of visibility improved the Cox and Snell $R_{\mathrm{pseudo}}^{2}$ from 0.165 in the exposure only model to 0.234.

**Figure 7:**
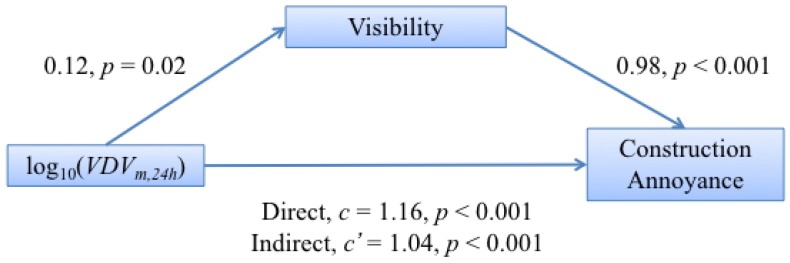
Schematic overview of the mediation model between vibration exposure, $\log_{10}\left(VDV_{m,24h}\right)$, and annoyance via visibility of the construction site. The values given are the estimated coefficients linking the two variables and their *p*-value significance.

From a preliminary multivariate analysis, there was some evidence of interaction between visibility and attitudinal factors. The interaction of visibility in two relationships was tested: vibration exposure predicting annoyance via visibility and exposure predicting concern via visibility. The former interaction was tested for moderation and found not to be significant (p=0.440). It was also tested for mediation, and all of the separate coefficients relating exposure, visibility and annoyance were significant (p<0.1). The direct path (*c*) between exposure and annoyance was larger than the indirect path ($c^{\prime }$) via visibility ($c-c^{\prime }=0.122$), and the Sobel test resulted in a p=0.028. This indicates that there was some mediation of visibility on the exposure-annoyance relationship. The pathway diagram with coefficients for each component for this mediation analysis is given in Figure 7.

**Figure 8:**
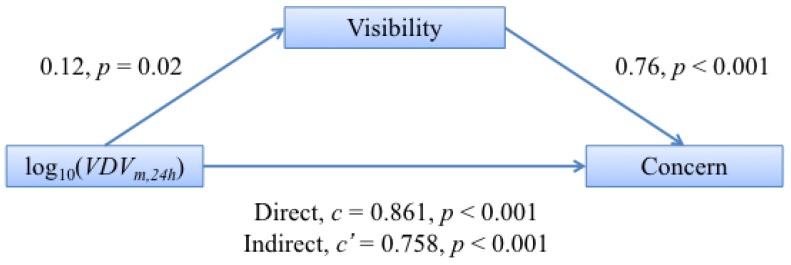
Schematic overview of the mediation model between vibration exposure, $\log_{10}\left(VDV_{m,24h}\right)$, and concern via visibility of the construction site. The values given are the estimated coefficients linking the two variables and their *p*-value significance.

Turning now to the latter interaction of visibility on the relationship between concern and vibration exposure for which no significant (p=0.633) moderation was found, all of the coefficients relating exposure, visibility and concern were found to be significant when testing for mediation, and the direct path was larger than the indirect path ($c-c^{\prime }=0.103$). The Sobel test resulted in p=0.035, indicating that visibility significantly mediates the annoyance from vibration and the concern from vibration. The mediation pathway diagram for this interaction is shown in Figure 8.

### 4.8. Time Spent at Home

In the questionnaire, respondents were asked to indicate during which three-hour blocks they were at home, and then, only the blocks corresponding to site hours were analysed. The number of hours a respondent was at home was split into five groups: 0, 1–3, 3–6, 6–9 and 9–12, covering 6 a.m.–6 p.m. There were 18 people who are not at home at all during those times.

Table 9 presents the parameter estimates from the ordinal logistic regression for high annoyance with the numbers of hours spent at home in the day time and exposure as a dependent variable. None of the coefficients for time spent at home were significant (p≫0.1), and there was no improvement in the model over exposure alone. Data were also collected about the number of hours spent at home during weekends, but again, this was not included in the analysis, as construction work did not take place on weekends [41].

### 4.9. Neighbourhood/Home Satisfaction

Two analyses were made predicting annoyance from firstly neighbourhood satisfaction and exposure (see Table 10) and secondly home satisfaction and exposure predicting annoyance (see Table 11).

From these data, it can be seen that for both cases, those who were more satisfied with their home or neighbourhood were less annoyed than those who were dissatisfied. It appears that satisfaction with the neighbourhood (β=0.580) has a stronger effect than satisfaction with the home (β=0.408) and also explains more of the variation in the annoyance data (neighbourhood Cox and Snell $R_{\mathrm{pseudo}}^{2}=0.254$; home Cox and Snell $R_{\mathrm{pseudo}}^{2}=0.197$).

### 4.10. Age

From a preliminary analysis, using age linearly, age was not found to significantly influence the annoyance from construction vibration. From an ordinal logistic regression with both exposure and age predicting annoyance, the Cox and Snell $R_{\mathrm{pseudo}}^{2}$ decreased from 0.165 down to 0.144, and the coefficients had a poor significance (p<0.1).

**Figure 9:**
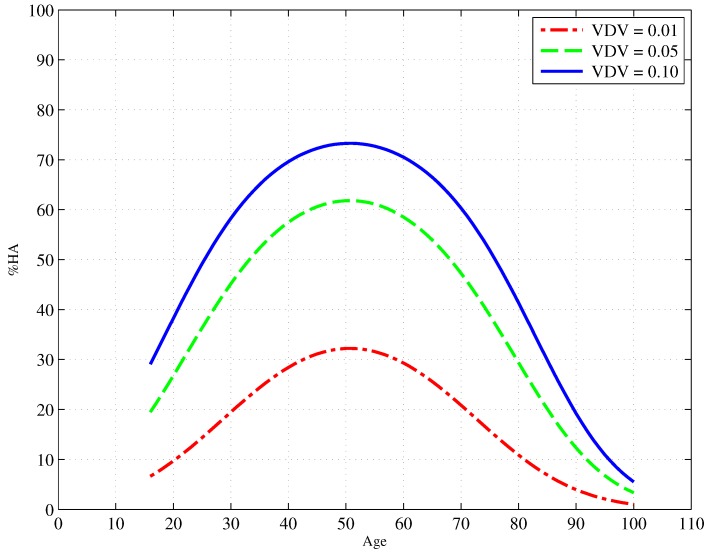
Exposure-response curves showing the proportion of people who are highly annoyed by construction vibration as a function of age for three different levels of vibration exposure ($VDV_{m,24h}m/s^{1.75}$).

**Figure 10:**
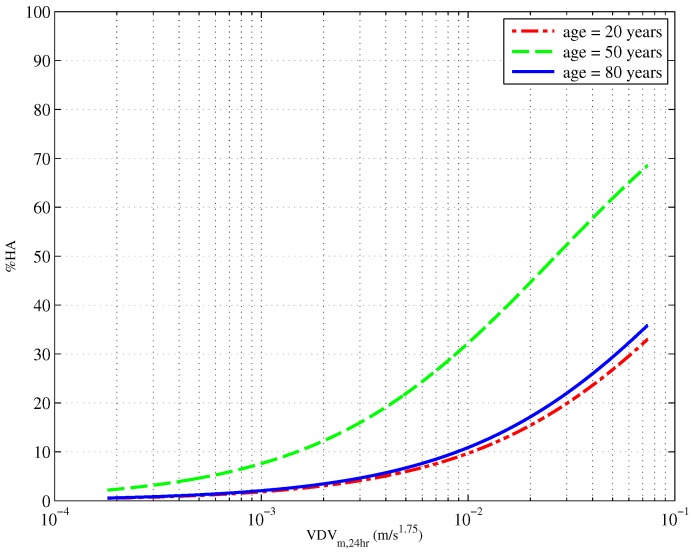
Exposure-response curves showing the proportion of people who are highly annoyed by construction vibration as a function of ${\mathrm{VDV}}_{m,24h}m/s^{1.75}$ for three different ages, 20, 50 and 80 years old.

Following the work of [32,47], an ordinal logistic regression was carried out with exposure, ${\left(\mathrm{age}/100\right)}^{2}$ and age/100 predicting annoyance. The results of this regression are presented in Table 12. The relationship between age and the annoyance of construction vibration is illustrated in two ways. Figure 9 and Figure 10 respectively present the proportion of people reporting as highly annoyed firstly across a range of ages and for three different vibration exposures and secondly across a range of vibration exposures and for three different ages (20, 50 and 80 years old).

The inclusion of age and ${\mathrm{age}}^{2}$ in the model yielded a small, but significant (p<0.001) improvement in the model raising the Cox and Snell $R_{\mathrm{pseudo}}^{2}$ from 0.165–0.193. Figure 9 shows an increase in annoyance for middle-aged people with a peak around 50 years of age. There is also an increase in high annoyance with increased exposure, and this can be seen in Figure 10. Again, it is the 50-year-olds who are most annoyed compared to both 20- and 80-year-olds.

### 4.11. Gender

Using ordinal logistic regression with gender and vibration exposure predicting annoyance, it was found that gender did not yield a significant (p≫0.1) improvement of the model over exposure alone. Table 13 presents the estimated parameters of the regression. From this, it can be seen that the coefficient for gender is not significant (p=0.125), and therefore, gender does not appear to have an important effect on the annoyance of construction vibration.

## 5. Discussion

### 5.1. Non-Exposure Factors

A comparison is made here to the work of Peris et al. [6] as it covers many of the same variables for railway vibration rather than construction vibration. It should be noted, however, that caution should be taken in drawing conclusions, as the vibration was also different. For this work, the range of ${\mathrm{VDV}}_{Wm}$ was from 0.00018–0.0739 ${m/s}^{1.75}$ with a mean of 0.022 ${m/s}^{1.75}$. For the railway vibration, the range was 0.00049–0.170 ${m/s}^{1.75}$ with a mean of 0.0179 ${m/s}^{1.75}$. Therefore, the level of exposure to construction vibration appears to be lower, but not too dissimilar.

#### 5.1.1. Self-Reported Sensitivity to Vibration

Self-reported sensitivity to noise was previously found to be important when understanding railway noise annoyance [13,14], but not for railway vibration annoyance [6]. For construction vibration, sensitivity does indeed significantly contribute to annoyance with the odds ratio suggesting a 2.3 increase in annoyance for every one point increase in sensitivity.

There is a potential effect of self-selection in making the comparison to the results of railway studies. The railway will have been in place for many tens of years, while the construction site will have been in place for a relatively short time. People moving into the area will have known that the railway was there and included that in their decision to move, but this will not have been the case for the construction. Respondents had to have been living in the property for at least nine months prior to the survey.

It is also important to be mindful of the effect that routing played in the questionnaire regarding self-reported sensitivity. Respondents who reported not feeling vibration nor its effects (e.g., rattling) were not then asked about their sensitivity. These ‘0’ responses for sensitivity were then recoded as “not at all” sensitive. This may lead to a general over estimation of sensitivity. Furthermore, the meaning of being sensitive to vibration is not widely understood in the way that noise sensitivity is.

#### 5.1.2. Expectation

For an exposure of ${\mathrm{VDV}}_{W_{m}}=0.01$ ${m/s}^{1.75}$, there was an increase of ∼11% in the proportion of respondents who reported being highly annoyed if they expected future vibration to worsen compared to those who thought it would stay the same or improve. Peris et al. [6] found a factor of three between the same two categories of people in response to railway vibration (${\mathrm{VDV}}_{W_{b}}=0.1$ ${m/s}^{1.75}$).

In general, those who expect that levels of future vibration will become worse are 1.8-times more likely to report a higher level of annoyance. Expectation did not, however, contribute greatly to the improvement of the model, as including it with exposure as a predictor of annoyance led to a decrease in the Cox and Snell pseudo $R^{2}$ compared to exposure alone.

An explanation for the difference between construction and railway vibration could be habituation and the temporary nature of construction. The railway, in use for tens of years, represents an almost permanent source of vibration unlike the construction site. An increase in vibration from the railway could represent a long-term change that the construction site may not. People would also know about the presence of the railway line prior to moving into a property and would therefore have considered their expectations of vibration and, to some degree, found it acceptable. Further analysis extending the comparison between railway and construction vibration using these data with a particular emphasis on the role of rattle is presented by Woodcock et al. [48].

#### 5.1.3. Concern about Property Damage

The results show that as vibration exposure is increased, the concern that residents have for property damage also increases, echoing the work of Peris et al. [6] for railway vibration. This is perhaps not surprising, as there is a logical link between greater vibration and the perceived likelihood of property damage. This overlaps with work on the effect of fear of transportation noise sources increasing annoyance [9,13,22]. Concern for property damage was found to both moderate and to mediate the relationship between exposure and annoyance. As with railway vibration, it was a partially mediating relationship suggesting that other mediational pathways were likely.

With specific regard to annoyance, it has been seen that concern for property damage has a wide ranging effects over a wide range of VDV. It is clear that concern for damage to property due to construction vibration is a factor that increases annoyance. As with sensitivity, the routing of the questionnaire may have played a role in this result, as those who did not report feeling vibration were not asked about their concern for property damage. They were then assumed not to be concerned, leading to a potential overestimation of concern. The levels of vibration that damage property are significantly higher than the levels at which some people become concerned. A way of reducing the annoyance felt by residents could be for information to be distributed, perhaps independently from the construction company, regarding levels of construction vibration and levels that are damaging.

#### 5.1.4. Tenure Type (Ownership)

The effect of ownership of a property was analysed in three ways: its influence on concern, its influence on annoyance and its interactions with those variables. Firstly, from an ordinal logistic regression using exposure and ownership as predictor variables, it was found that owners were more likely to be annoyed than renters with an odds ratio of 2.7. This supports the work of Peris et al. [6] who found a similar result for railway vibration, but contrasts with the findings of Miedema and Vos [13] who found no link for transportation noise.

From the ordinal logistic regression of concern predicted by ownership, it was found that owners were 4.1-times more likely to report a higher level of concern than renters. This was far larger than the 1.6-times found for railway vibration. As with expectation, this could be because people are likely to have taken up residence with knowledge of the railway unlike the temporary construction site.

With regard to interactions, it was found that there was no moderation of ownership on the exposure-annoyance relationship, but that there was a partial mediation of it. Further, ownership partially mediates the relationship between exposure and concern. This suggests some sort of chain: exposure, ownership, concern, then annoyance. None of the mediating links between these variables was total, and therefore, other pathways might be expected; but, there is apparently logical sense to these findings. The amount that residents are annoyed depends somewhat on how concerned they are about their property being damaged by the vibration, and the extent to which they are concerned is partially explained by whether they own the property or not. Those who do not own the property are less financially impacted by damage and, therefore, less concerned or annoyed.

#### 5.1.5. Visibility

An odds ratio of 3.3 was found between the annoyance reported by those who could see the construction site from the residence compared to those who could not, with those able to see it being more annoyed. This is in line with the results for railway noise [29,30] and for railway vibration [6]. The interaction between exposure and visibility was tested for mediation in predicting annoyance and in predicting concern. It was found that visibility does indeed partially mediate both the relationship between exposure and annoyance and the relationship between exposure and concern. That visibility mediates between exposure and annoyances means that, to some extent, greater vibration means a greater chance of visibility. This is logical given that those closer to the site will have both greater exposure and more chance of being able to see the site. Similarly, proximity to the site may increase people’s concerns about property damage.

#### 5.1.6. Age

Age has previously been found to correlate with noise annoyance, but not so for other demographic factors [10,13]. An inverted “U” shape relationship between age and annoyance was previously reported for railway vibration [6] with those in their middle age (around 45) reporting the highest levels of annoyance. This supported other work [32], and a similar result was found in this work for vibrations from construction sites. Fifty-year-olds were the most likely to be annoyed, with both younger and older people being less annoyed. For all ages, annoyance increases with exposure. No explanation is yet certain, but it is posited that people of middle age tend to be home owners; therefore, this “U” shaped result could be overlapping with previous findings of ownership, concern and annoyance.

#### 5.1.7. Gender

Gender was not found to significantly improve the exposure-response models for railways, and a similar finding was made here. It appears that men and women react in a similar way with respect to the annoyance from construction vibration.

#### 5.1.8. Home/Neighbourhood Satisfaction

From an ordinal logistic regression, it was found that those who were more satisfied with their homes or neighbourhood tended to be less annoyed about construction vibration than those who were dissatisfied. On the five-point scale very satisfied to very dissatisfied, a one point increase in neighbourhood dissatisfaction would lead to a 1.5-times increase in the probability of reporting a higher level of annoyance with a 1.8-times increase for home dissatisfaction. This result reflects the findings on railway vibration annoyance [6].

### 5.2. Noise Exposure

There were a number of practical issues encountered in obtaining noise exposures from construction sources. When attempting to measure internal noise exposures, the construction noise of interest was often masked by extraneous internal sources. Consequently, poor measurements of many events or no events could be obtained internally. External measurements yielded clearer and distinct events. However external noise measurements were often influenced by background noise, particularly road traffic.

Limitations were also encountered due to problems of installing the equipment. Little or no surrounding space remained for public access and, thus, for finding a receiver position in the vicinity of the construction area. One of the biggest challenges found during measurements was to anticipate the schedule of work on construction sites and obtaining data from measurements of as many representative noise sources as possible. Due to its nature, construction work, regardless of the organisation of the site, could not be accurately predicted. As a consequence of this, a small number of processes were occasionally missed and measurement data not obtained.

The noise exposures from the construction sites were seen to differ slightly. This is explained by the increased distance to facades, over 100 m in some cases, thus limiting reflective effects. The distance itself causes a significant reduction in noise level, although other buildings could have an observable impact on the final results by partially obscuring sources. On the other hand, construction work at some sites was essentially situated within residential areas. In fact, a great number of residents were located directly alongside the main road upon which the construction was sited and direct exposure to the resulting noise is therefore higher.

Unfortunately, despite the success of the efforts taken in the design of the questionnaire, it was not be possible to separate the annoyance due to construction noise from the proportion due to vibration. This is because both noise and vibration exposure estimation procedures involved extrapolation from measurements at a reference point, which means that they correlate as mentioned earlier. Furthermore, noise measurements were significantly contaminated by background noise, particularly by road traffic. These are important considerations for other researchers when planning and designing similar studies.

Future measurements could consider a much more sophisticated approach, e.g., as performed by Ballesteros [49]. Three suggestions were made as being of greatest importance:

- A weekly continuous monitoring of all construction work stages for longer periods of time;
- Recording of sound during monitoring so that sources could be identified and easily recognised;
- Possibility of setting a measurement instrument within the vicinity of a source or installing a number of instruments, such that a construction process will occur within close proximity of at least one.

One of the direct consequences of the improved methodology is that associated uncertainties from measurement can be significantly reduced due to better identification of a source, decreased influence from background noise and a longer period of measurement time, ensuring that all noise events are recorded.

### 5.3. Implications

The main implication of these results is that in some situations, the most cost-effective way to reduce annoyance is to address attitudinal factors, such as concern, rather than to put in place measures to reduce vibration exposure. Attitudinal factors could be addressed for example by community meetings, particularly with regard to keeping residents informed about future vibration levels. In practice, a combined approach is likely to be required, considering vibration mitigation and non-exposure factors based on a cost-benefit analysis.

## 6. Conclusions

A range of non-exposure factors have been investigated with the aim of determining whether they influence the extent to which nearby residents are annoyed by vibration from sites of LRT construction. Of the three categories, attitudinal, situational and demographic, it was the attitudinal factors that were found to influence the exposure-annoyance relationship the most.

Concern for property damage had the greatest influence of the attitudinal factors, with greater concern leading to a greater probability of reporting as highly annoyed. Concern also appeared to mediate in the relationship between vibration and annoyance. Further to this, it was found that ownership of the residence increased the concern of respondents. Sensitivity to vibration was a more significant factor than expectation of future vibration.

Of the situational factors, the visibility of the construction site had the most influence over the people’s annoyance from vibration. Those who could see the construction site were more likely to be annoyed, and visibility also mediated both the exposure-annoyance and exposure-concern relationships. Of the demographic factors explored, only age was found to have an impact on annoyance, with an inverted “U” relationship showing that those of middle age were the most annoyed.

These findings bear similarity to those for railway vibration and noise, particularly as attitudinal variables were important factors for both and, in particular, concern. Ownership of the property was also an important factor for both railway vibration and construction. A similar “U”-shaped relation between age and annoyance was found, as well.

These findings bear similarity to those for railway vibration and noise, particularly as attitudinal variables were important factors for both and, in particular, concern. Ownership of the property was also an important factor for both railway vibration and construction. A similar “U”-shaped relation between age and annoyance was found, as well. The results indicate that when policy is prepared regarding the impact on residents of building a light rapid transit system, attitudinal factors should be considered carefully.

## Figures and Tables

**Table 1:** Age distribution of respondents. Thirty two ages were not reported and are not included in the table.

**Age Range**	16–25	26–35	36–45	46–55	56–65	66–75	76–85	>85
**Frequency**	36	59	68	50	48	37	18	2

**Table 2:** Estimated parameters for the exposure-response of construction vibration and annoyance. This table is reproduced with permission from [7].

Parameter	Estimates			
	Estimates	SE	95% CI	
			Lower	Upper
Threshold (*τ*)				
Not at all	−3.153	0.477	−4.088	−2.218
Slightly	−2.731	0.469	−3.649	−1.812
Moderately	−2.177	0.460	−3.079	−1.276
Very	−1.599	0.456	−2.493	−0.704
Location (*β*)				
log_10_ VDV*_m,24h_*	1.771	0.254	1.273	2.269

Link function: Logit. All coefficients were significant (p<0.001). Cox and Snell $R_{\mathrm{pseudo}}^{2}=0.165$, Nagelkerke $R_{\mathrm{pseudo}}^{2}=0.178$.

**Table 3:** Estimated parameters for the exposure-response of construction vibration with self-reported sensitivity as a factor.

Parameter Estimates	Estimates			
	Estimates	SE	95% CI	
			Lower	Upper
Threshold (*τ*)				
Highly Annoyed	1.046 *	0.588	−0.106	2.197
Location ($\beta_{0}$)				
log_10_ VDV*_m,24h_*	0.908 ***	0.261	0.397	1.419
Sensitivity ($\beta_{1}$)	0.845 ***	0.114	0.622	1.068

Link function: Logit. *n* = 250, * p<0.1, *** p<0.001. Cox and Snell $R_{\mathrm{pseudo}}^{2}=0.316$, Nagelkerke $R_{\mathrm{pseudo}}^{2}=0.333$.

**Table 4:** Estimated parameters for the exposure-response of annoyance of construction vibration with exposure and concern for property damage as predicting factors.

Parameters	Estimates			
	Estimates	SE	95% CI	
			Lower	Upper
Threshold (*τ*)				
Highly Annoyed	1.504 **	0.569	0.388	2.619
Location (*β*)				
log_10_ VDV*_m,24h_*	1.243 ***	0.270	0.713	1.773
Concern	1.032 ***	0.094	0.847	1.217

Link function: Logit. ** p<0.01, *** p<0.001. Cox and Snell $R_{\mathrm{pseudo}}^{2}=0.463$, Nagelkerke $R_{\mathrm{pseudo}}^{2}=0.499$. n=321.

**Table 5:** Estimated parameters for the exposure-response of construction vibration with expectations of future vibration as a factor.

Parameters	Estimates			
	Estimates	SE	95% CI	
			Lower	Upper
Threshold (*τ*)				
Highly Annoyed	−1.148 *	0.503	−2.134	−0.163
Location (*β*)				
log_10_ VDV*_m,24h_*	1.213 ***	0.253	0.716	1.710
Expectation	0.567 *	0.245	0.086	1.048
(vibrations will worsen)				

Link function: Logit. n=248. Cox and Snell $R_{\mathrm{pseudo}}^{2}=0.133$, Nagelkerke $R_{\mathrm{pseudo}}^{2}=0.14$. * p<0.1, *** p<0.001.

**Table 6:** Estimated parameters for the exposure-response of annoyance from construction vibration with property ownership as a factor.

Parameters	Estimates			
	Estimates	SE	95% CI	
			Lower	Upper
Threshold (*τ*)				
Highly Annoyed	−2.451 ***	0.481	−3.394	−1.508
Location (*β*)				
log_10_ VDV*_m,24h_*	1.782 ***	0.263	1.267	2.297
Rent	−1.000 ***	0.261	−1.511	−0.489

Link function: Logit. n=319. Cox and Snell $R_{\mathrm{pseudo}}^{2}=0.202$, Nagelkerke $R_{\mathrm{pseudo}}^{2}=0.218$. *** p<0.001.

**Table 7:** Estimated parameters for the exposure-response of concern for property damage from construction vibration with property ownership as a factor.

	Estimates			
Parameters	Estimates	SE	95% CI	
			Lower	Upper
Threshold (*τ*)				
High Concern	−1.530 **	0.457	−2.426	−0.635
Location (*β*)				
log_10_ VDV*_m,24h_*	1.340 ***	0.245	0.859	1.820
Rent	−1.402 ***	0.267	−1.925	−0.879

Link function: Logit. n=319. Cox and Snell $R_{\mathrm{pseudo}}^{2}=0.193$, Nagelkerke $R_{\mathrm{pseudo}}^{2}=0.207$. ** p<0.01, *** p<0.001.

**Table 8:** Estimated parameters for the exposure-response of annoyance from construction vibration with visibility of the construction site as a factor.

	Estimates			
Parameters	Estimates	SE	95% CI	
			Lower	Upper
Threshold (*τ*)				
Highly Annoyed	−1.803 ***	0.453	−2.692	−0.915
Location (*β*)				
log_10_ VDV*_m,24h_*	1.546 ***	0.246	1.064	2.028
Visibility (not visible)	−1.207 ***	0.233	−1.662	−0.751

Link function: Logit. Cox and Snell $R_{\mathrm{pseudo}}^{2}=0.234$, Nagelkerke $R_{\mathrm{pseudo}}^{2}=0.252$. *** p<0.001.

**Table 9:** Estimated parameters for the exposure-response of annoyance from construction vibration with the amount of time spent at home between 06:00 and 18:00 on weekdays as a factor.

	Estimates			
Parameters	Estimates	SE	95% CI	
			Lower	Upper
Threshold (*τ*)				
High	−2.174 ***	0.473	−3.101	−1.246
Location (*β*)				
log_10_ VDV*_m,24h_*	1.731 ***	0.259	1.224	2.238
0 h	−0.538	0.542	−1.599	0.524
1–3 h	−0.255	0.317	−0.877	0.367
3–6 h	0.442	0.440	−0.421	1.305
6–9 h	−0.218	0.533	−1.262	0.826

Link function: Logit. n=303. Cox and Snell $R_{\mathrm{pseudo}}^{2}=0.167$, Nagelkerke $R_{\mathrm{pseudo}}^{2}=0.180$. *** p<0.001.

**Table 10:** Estimated parameters for the exposure-response of annoyance from construction vibration with neighbourhood satisfaction as an independent variable.

	Estimates			
Parameters	Estimates	SE	95% CI	
			Lower	Upper
Threshold (*τ*)				
Highly Annoyed	−0.690	0.518	−1.704	0.325
Location (*β*)				
log_10_ VDV*_m,24h_*	1.663 ***	0.256	1.161	2.165
Neighbourhood Satisfaction	0.580 ***	0.098	0.389	0.772

Link function: Logit. n=321. Cox and Snell $R_{\mathrm{pseudo}}^{2}=0.254$, Nagelkerke $R_{\mathrm{pseudo}}^{2}=0.274$. *** p<0.001.

**Table 11:** Estimated parameters for the exposure-response of annoyance from construction vibration with home satisfaction as an independent variable.

	Estimates			
Parameters	Estimates	SE	95% CI	
			Lower	Upper
Threshold (*τ*)				
Highly Annoyed	−1.441 **	0.502	−2.424	−0.457
Location (*β*)				
log_10_ VDV*_m,24h_*	1.794 ***	0.257	1.292	2.297
Home Satisfaction	0.408 ***	0.114	0.185	0.630

Link function: Logit. n=321. Cox and Snell $R_{\mathrm{pseudo}}^{2}=0.197$, Nagelkerke $R_{\mathrm{pseudo}}^{2}=0.212$. ** p<0.01, *** p<0.001.

**Table 12:** Estimated parameters for the exposure-response of annoyance from construction vibration with “age” and “${\mathrm{age}}^{2}$ as independent variables.

	Estimates			
Parameters	Estimates	SE	95% CI	
			Lower	Upper
Threshold (*τ*)				
Highly Annoyed	1.298	0.978	−0.619	3.215
Location (*β*)				
log_10_ VDV*_m,24h_*	1.753 ***	0.269	1.225	2.281
Age/100	−16.027 ***	4.090	−24.043	−8.011
(Age/100)^2^	15.815 ***	4.064	7.850	23.780

Link function: Logit. n=294. Cox and Snell $R_{\mathrm{pseudo}}^{2}=0.193$, Nagelkerke $R_{\mathrm{pseudo}}^{2}=0.208$. *** p<0.001.

**Table 13:** Estimated parameters for the exposure-response of annoyance from construction vibration with property gender as a factor.

	Estimates			
Parameters	Estimates	SE	95% CI	
			Lower	Upper
Threshold (*τ*)				
High	−1.782 ***	0.475	−2.712	−0.852
Location (*β*)				
log_10_ VDV*_m,24h_*	1.801 ***	0.256	1.299	2.304
Male	−0.356	0.232	−0.811	0.099

Link function: Logit. n=294. Cox and Snell $R_{\mathrm{pseudo}}^{2}=0.171$, Nagelkerke $R_{\mathrm{pseudo}}^{2}=0.184$. *** p<0.001.

## Appendix A. A Questionnaire

**Table A1:** Summary of responses to relevant survey questions. Information regarding any recoding is also provided along with relevant routing within the questionnaire.

Question	Responses	Frequency	%	Total/350	Recoding/Routing
A1. In which of the following is the property situated?	Centre of a large city	0	0.0	336	
	Suburbs/Outskirts of a large city	257	73.4		
	Large town or small city	76	21.7		
	Small town	3	0.9		
	Village	0	0.0		
	Countryside	0	0.0		
	Other	0	0.0		
	Missing	14	4.0		
B4. Looking at this card [show card 1], overall, how satisfied or dissatisfied are you personally with living in this neighbourhood? Would you say that you are very satisfied, satisfied, neither satisfied nor dissatisfied, dissatisfied or very dissatisfied?	Very satisfied	112	32.0	350	
	Satisfied	160	45.7		
	Neither satisfied nor dissatisfied	25	7.1		
	Dissatisfied	27	7.7		
	Very dissatisfied	26	7.4		
	Missing	0	0.0		
C2. Looking at this card [show card 1], overall, how satisfied or dissatisfied are you personally with living in this home? Would you say that you are very satisfied, satisfied, neither satisfied nor dissatisfied, dissatisfied or very dissatisfied?	Very satisfied	138	39.4	350	
	Satisfied	163	46.6		
	Neither satisfied nor dissatisfied	21	6.0		
	Dissatisfied	16	4.6		
	Very dissatisfied	12	3.4		
	Missing	0	0.0		
C9. Looking at this list [show card 3], which best describes your current situation with this home? Do you or your family:	Own outright or with a mortgage	228	65.1	348	Responses to this questions were recoded as to whether they had some stake in the property or not, i.e., A: (Own/Mortgage/Part mortgage), B: (Private Rent/Association Rent).
	Part-rent and part-own with a mortgage	9	2.6		
	Rent from a private landlord/letting agency	53	15.1		
	Rent from a Housing Association or Council	53	15.1		
	Other	5	1.4		
	Missing	2	0.6		
C12. From any room in this home, can you see: Construction Activity	No	202	57.7	345	
	Yes	143	40.9		
	Missing	5	1.4		

**Table A2:** Response summary continued.

Question	Responses	Frequency	%	Total/350	Recoding/Routing
D1. Thinking about the time you have been living here, when indoors at home, have you felt any vibration or shaking anywhere that you think was caused by:	Cars, lorries, buses and other road vehicles	N: 229, Y: 120	N: 65.4, Y: 34.4	349	If respondents answered yes to any of these they were directed to further questions (D2–D4) about the vibration they felt: How, Where, Activity disturbed. Else they moved to questions about rattle.
	Aeroplanes	N: 343, Y: 7	N: 98.0, Y: 2.0	350	
	Helicopters	N: 340, Y: 38	N: 97.1, Y: 2.9	350	
	Railway activity	N: 311, Y: 38	N: 88.9, Y: 10.9	349	
	Underground trains like the tube or metro	N: 343, Y: 6	N: 98.0, Y: 1.7	349	
	Trains in tunnels	N: 345, Y: 6	N: 98.0, Y: 1.7	349	
	Construction activity, including demolition, piling, road works, drilling, surface activity such as bulldozers and loading trucks and any other construction activity	N: 115, Y: 235	N: 32.9, Y: 67.1	350	
	Quarrying or mining	N: 348, Y: 2	N: 99.4, Y: 0.6	350	
	Footsteps, slamming doors, domestic appliances inside this home	N: 330, Y: 20	N: 94.3, Y: 5.7	350	
	Footsteps, slamming doors, domestic appliances in neighbouring homes	N: 338, Y: 12	N: 96.6, Y: 3.4	350	
	An unidentified source	N: 349, Y: 1	N: 99.7, Y: 0.3	350	
	Any other source	N: 336, Y: 14	N: 96.0, Y: 4.0	350	
	Summary, Felt vibration at all	N: 93, Y: 256	N: 26.6, Y: 73.3	349	
D5. Thinking about the time you have been living here, when indoors at home, have you heard or seen things rattle, vibrate or shake that you think was caused by:	Cars, lorries, buses and other road vehicles	N: 286, Y: 55	N: 81.7, Y: 15.7	341	If respondents answered yes to any of these they were directed to further questions (D6–D8) about the rattle they felt: How, Where, Activity disturbed.
	Aeroplanes	N: 340, Y: 1	N: 97.1, Y: 0.3	341	
	Helicopters	N: 337, Y: 4	N: 96.3, Y: 1.1	341	
	Railway activity	N: 319, Y: 22	N: 91.1, Y: 6.3	341	
	Underground trains like the tube or metro	N: 338, Y: 3	N: 96.6, Y: 0.9	341	
	Trains in tunnels	N: 340, Y: 1	N: 99.7, Y: 0.3	341	
	Construction activity, including demolition, piling, road works, drilling, surface activity such as bulldozers and loading trucks and any other construction activity	N: 182, Y: 159	N: 53.4, Y: 46.6	341	
	Quarrying or mining	N: 340, Y: 1	N: 99.7, Y: 0.3	341	
	Footsteps, slamming doors, domestic	N: 335, Y: 6	N: 95.7, Y: 1.7	341	
	appliances inside this home				
	Footsteps, slamming doors, domestic appliances in neighbouring homes	N: 335, Y: 6	N: 95.7, Y: 1.7	341	
	An unidentified source	N: 341, Y: 0	N: 100	341	
	Any other source	N: 336, Y: 5	N: 96.0, Y: 1.4	341	
	Summary, Saw rattle at all	N: 167, Y: 174	N: 49.0, Y: 51.0	341	
Summary of Vibration and Rattle	Felt or saw either vibration or rattle at all	N: 84, Y: 266	N: 24.0, Y:76.0	350	

**Table A3:** Response summary continued.

Question	Responses	Frequency	%	Total/350	Recoding/Routing
D9. Thinking about the time you have been living here, when indoors at home, how bothered, annoyed or disturbed have you been by feeling vibration or shaking or hearing or seeing things rattle, vibrate or shake caused by construction activity?	don’t feel	120.0	34.3	350	Respondents were asked about their annoyance for the vibration sources they had noticed. If respondents previously stated they did not notice the vibration they were classed as don’t feel and then later recoded as not at all.
	not at all	66.0	18.9		
	slightly	32.0	9.1		
	moderately	38.0	10.9		
	very	31.0	8.9		
	extremely	63.0	18		
D11. In the future, do you think the level of vibration you experience whilst indoors at home will get worse, get better or remain the same?	Worse	71	20.3	273	Responses to this question were later recoded into two groups: A (Worse and Don’t Know) and B (Same and Better). Only those reporting noticing vibration or rattle were asked this question.
	Better	81	23.1		
	Same	43	12.3		
	Don’t Know	78	22.3		
	Missing	77	22.0		
D13. We would like to know if you are concerned that the vibration may damage this home or your possessions inside it in any way.	not at all	181	51.7	350	Only those reporting noticing vibration or rattle were asked this question. If respondents previously stated they did not notice vibration nor rattling they were re-classed as not at all.
	slightly	43	12.3		
	moderately	41	11.7		
	very	39	11.1		
	extremely	46	13.1		
	Missing	0	0.0		
D15. How sensitive would you say you are personally to vibration in general? Would you say you are not at all sensitive, slightly sensitive, moderately sensitive, very sensitive or extremely sensitive?	not at all	98	28.0	273	If respondents previously stated that they did not notice vibration nor rattling they were not asked this questions and were re-coded as not at all.
	slightly	78	22.3		
	moderately	51	14.6		
	very	27	7.7		
	extremely	19	5.4		
	Missing	77	22.0		
Y1. During a typical weekday, that is, Monday to Friday, what times are you usually at home? Are you at home between:	06:01 and 09:00	301	86.0	330	
	09:01 and 12:00	240	68.6		
	12:01 and 15:00	234	66.9		
	15:01 and 18:00	249	71.1		
	18:01 and 21:00	307	87.7		
	21:01 and 00:00	316	90.3		
	00:01 and 03:00	321	91.7		
	03:01 and 06:00	322	92.0		
	Missing	20	5.7		
Y2. During a typical weekend, that is, Saturday and Sunday, what times are you usually at home? Are you at home between:	06:01 and 09:00	321	91.7	330	
	09:01 and 12:00	301	86.0		
	12:01 and 15:00	288	82.3		
	15:01 and 18:00	293	83.7		
	18:01 and 21:00	305	87.1		
	21:01 and 00:00	311	88.9		
	00:01 and 03:00	315	90.0		
	03:01 and 06:00	320	91.4		
	Missing	19	5.4		
